# Pulsed laser deposition of Zn(O,Se) layers in nitrogen background Pressure

**DOI:** 10.1038/s41598-019-54008-1

**Published:** 2019-11-25

**Authors:** Akram Abdalla, Sergei Bereznev, Nicolae Spalatu, Olga Volobujeva, Natalja Sleptsuk, Mati Danilson

**Affiliations:** 1 School of Engineering, Department of Materials and Environmental Technology, Tallinn University of Technology, Ehitajate tee 5, Tallinn, 19086 Estonia; 2 TJS Department of Electronics, Tallinn University of Technology, Ehitajate tee 5, Tallinn, 19086 Estonia

**Keywords:** Energy science and technology, Materials science

## Abstract

Zinc oxy-selenide Zn(O,Se) is a novel material, that can replace the toxic CdS buffer layer in thin film solar cells and other optoelectronic devices. In this paper a systematic study of the structural, optical and electrical properties of Zn(O,Se) layers, grown by pulsed laser deposition under 50 mTorr of nitrogen background pressure, over a wide range of the substrate temperature, from RT to 600 °C, is reported. XRD, Raman, HR-SEM, XPS, UV-Vis techniques and Hall effect measurements have been used to investigate the structural, and optoelectronic properties of Zn(O,Se) layers. XRD analysis revealed that the polycrystalline ternary Zn(O,Se) phase formed at 500 °C. Raman analysis confirmed the formation of the polycrystalline Zn(O,Se) phase at 500 °C and an amorphous phase at substrate temperatures below 500 °C. Similarly, XPS analysis accompanied with the modified Auger parameters confirmed formation of ternary Zn(O,Se) layer at 500 °C as well. HR-SEM investigation showed the growth of homogenous, dense and adherent films onto a glass substrate. Furthermore, optical studies revealed that all prepared films are practically transparent in the visible region of the spectrum, with a band gap around 3 eV. Hall effect measurements revealed that conductivity, and electron concentration, increased by four orders of magnitude at 600 °C. It was found, that nitrogen background pressure maintained stable ratios of elemental contents in the whole range of the substrate temperature for Zn(O,Se) layers.

## Introduction

An urgent global need for sustainable energy resources has opened a multi-disciplinary research area towards potential clean energy resources, such as solar cells and hydrogen generation^1^, as renewable energy sources. With respect to solar cells, particularly in high efficiency thin film solar cells such as CdTe and CIGS, a thin layer of CdS is used as a standard buffer layer which has, up to now, achieved a power conversion efficiency of 12.6%, 22.1% and 23.35% for CZTS^2,3^, CdTe^2,4^ and CIGS^2,5^ solar cells respectively. On the other hand, CdS is a toxic material having parasitic absorption in the 350–550 nm range, which decrease the efficiency of the device^6–8^. To address this issue, significant attention has been devoted to replacing the CdS buffer layer. Thus, synthesis of new, wide band gap, buffer material, with optimized optical and electrical properties, to overcome the CdS limitations, is required. As a result, different materials have been tested and reported such as Zn(O,OH)_x_/Zn(O,S,OH)_x_^9^, Zn_1-x_Mg_x_O^10,11^, ZnS/ZnSe^12^, indium sulfide/indium selenide^13,14^ and ZnO^15^.

Zinc oxy-selenide is an excellent candidate for the substitution of the CdS buffer layer in thin film solar cell structure due to its non-toxic, earth abundant and tuneable wide band gap character^16^. On the other hand, there is limited information available about the structural, optical and electrical properties of this material. Zn(O,Se) layers can be prepared by different methods such as pulsed laser deposition (PLD)^1,16^, radio frequency (RF) sputtering^17^ and molecular beam epitaxy (MBE)^18^. Among them, PLD is one of the most promising techniques for the stoichiometric deposition of complex-oxide heterostructures, super-lattices, and well-controlled interfaces^19^. High quality, uniform and adherent layers can be deposited by PLD.

Recently, band gap grading, via incorporation of selenium in the CdTe absorber has improved the overall cell efficiency^8,20–22^. For instance, CdSe, used as partner buffer layer for CdTe solar cells, instead of CdS, improved cell performance and recorded a power conversion efficiency of 14% with low open-circuit-voltage (Voc) and fill factor (FF), but high short circuit current (Jsc)^8,23^. It was demonstrated that during the cell processing, CdSe diffused into CdTe, converting itself from photo-inactive CdSe to photo-active, low band gap, CdTe_1-x_Se_x_^23,24^ with the successful removal of the undesired CdSe sub-layer. In other words, this approach extended photon collection to a longer wavelength, reduced losses in short wavelength and increased the carrier lifetime so giving excellent device performance^22,25^. With regards to this, in previous work^16^, ternary Zn(O,Se) thin film was deposited by PLD in high vacuum. Its structural, optical and electrical properties were investigated and Zn(O,Se) layers were applied, for the first time, as a buffer layer, with CdTe absorber in a superstrate structure which yielded a reasonable power conversion efficiency of 7.6%^16^. It was also found that the crystal structure of Zn(O,Se) film only formed at 500 °C, and all structures deposited below this growth temperature were amorphous. Herein, this view is extended to control the composition contents and to develop the crystal structure of Zn(O,Se) layers by depositing it in nitrogen background pressure instead of high vacuum. For composition control, the nitrogen back-pressure reduce the mean free path and kinetic energy of the ablated particles. Which it has an impact of retaining the ablated particles and prevent their evaporation from the substrate surface. In case of crystal structure, the nitrogen partial pressure effects the lattice parameters of the films which will be reflected in the crystal structure^26,27^.

The aim of this work was the deposition of Zn(O,Se) thin films by PLD under nitrogen back-pressure and different substrate temperatures (Ts), with the study of the structural and optoelectronic properties of the prepared Zn(O,Se) layers. Special attention was paid to the comparison between Zn(O,Se) layers deposited in high vacuum and under nitrogen back-pressure.

## Results and Discussions

**Figure 1:**
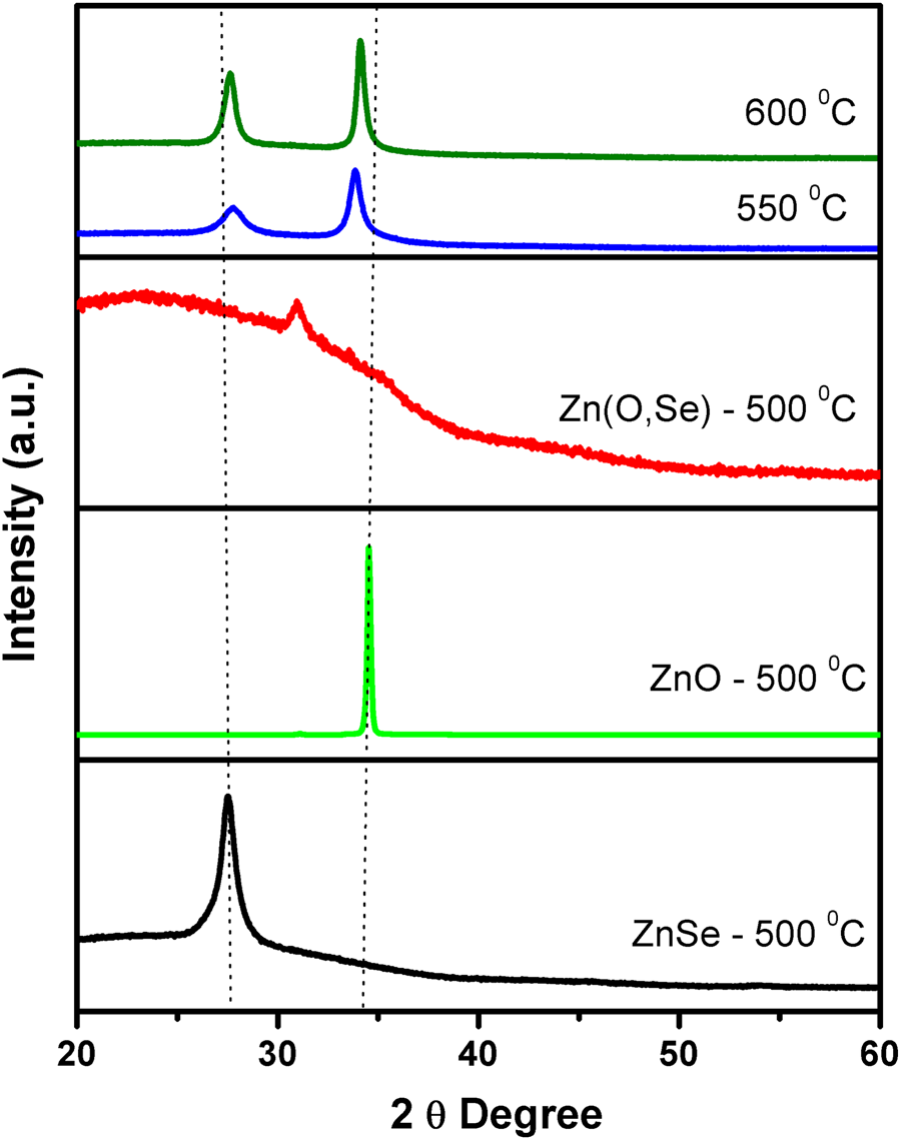
XRD patterns of the Zn(O,Se) films deposited in the substrate temperature region of 500–600 °C under nitrogen back-pressure.

According to XRD results all Zn(O,Se) layers deposited below 500 °C were amorphous (see Supplementary Fig. S1). Figure 1 represents the XRD patterns of the Zn(O,Se) layers deposited at the substrate temperature from 500 to 600 °C combined with the XRD patterns of pure phases of ZnO and ZnSe layers deposited at 500 °C for comparison. The ZnO layer showed an XRD peak at 34.53°. In the literature, this peak is assigned to (002) plane of the ZnO wurtzite structure^28–34^, while the ZnSe layer exhibited an XRD peak at 27.47° which is well indexed to (111) plane of ZnSe cubic phase^35,36^. Zn(O,Se) film, grown at 500 °C, showed only one diffraction peak at 30.94° together with a residual amorphous hump, attributed to the formation of an oriented polycrystalline Zn(O,Se) phase mixed with an amorphous phase. The diffraction peak located at approximately 30.94° has been shown, in the literature, as corresponding to (002) plane ZnO_1-x_Se_x_ in which the x values varied from 0 to 0.12^1^. As the substrate temperature was increased to 550 °C the diffractogram showed two distinct peaks at 27.70° and 34.09° indicating to the formation of different solid solutions. These peaks cannot be assigned to pure phases of ZnO and ZnSe as their diffractograms showed reflections at different 2θ values/positions. Considering the solubility of oxygen in ZnSe and selenium in ZnO, the XRD peak located at 27.7° and 30.09° can be attributed to the formation of ZnSe(_1-*y*_)O_*y*_ and ZnO(_1-*z*_)Se_*z*_ solid solutions, where *y* and *z* ≪ 1 indicates the low solubility of O and Se in ZnSe and ZnO, respectively. With increasing the substrate temperature to 600 °C, both peaks shift towards 2θ values of ZnO and ZnSe pure phases, indicating to further decrease of Se and O solubility in ZnO and ZnSe, correspondingly.

Furthermore, an observable increase in both XRD peak intensities has been noticed with increasing the substrate temperature from 500 to 600 °C, and this behaviour is attributed to an increase in the crystallite size (see Table 1). The Scherrer formula (1) was used to calculate the crystallite size *D*^37^.

1 $$D=\frac{0.94\lambda }{\beta cos\theta }$$

where λ is wavelength of X-ray radiation (1.5406 Å), θ is the Bragg angle and β is the full width at the half maximum (FWHM) of the peak located in the 2θ at 30.94–34.09° range in radians.

**Table 1:** The crystallite sizes, elemental contents, film thicknesses and band gap values (*E*_*g*_) of the Zn(O,Se) films deposited at 500–600 °C under nitrogen back-pressure.

Ts, (°C)	Crystallite sizes (nm)^a^	Elemental ratios (at%)^b^			Film Thickness (nm)	Band gap (eV)
		Zn	O	Se		
500	12.60 ± 0.8	50	39	11	454	3.00
550	13.40 ± 0.13	50	37	13	484	2.90, 3.28
600	20.50 ± 0.9	50	37	13	459	3.16, 3.29

The error bars in ^a^represent standard deviation (s.d.).

^b^Measurement error of EDX is ±0.5.

The crystallite sizes of Zn(O,Se) films deposited at 500–600 °C is calculated from the peak located at 30.94–34.09° and summarized in Table 1. The crystallite sizes increased with the increase of the substrate temperature. In comparison with PLD deposited Zn(O,Se) films in high vacuum^16^, the crystallite sizes decreased for the similar growth temperature interval. This can be due to an effect of the nitrogen background pressure. The nitrogen background pressure increases the nucleation sites because of the increased reaction of the ablated particles at the surface^27^. Therefore, the surface diffusion length of atoms is decreased. In addition, the kinetic energy of the ablated species is decreased by increasing the number of collisions between the ablated species in the gas phase. As a result, the crystallite size decreases^27^.

**Figure 2:**
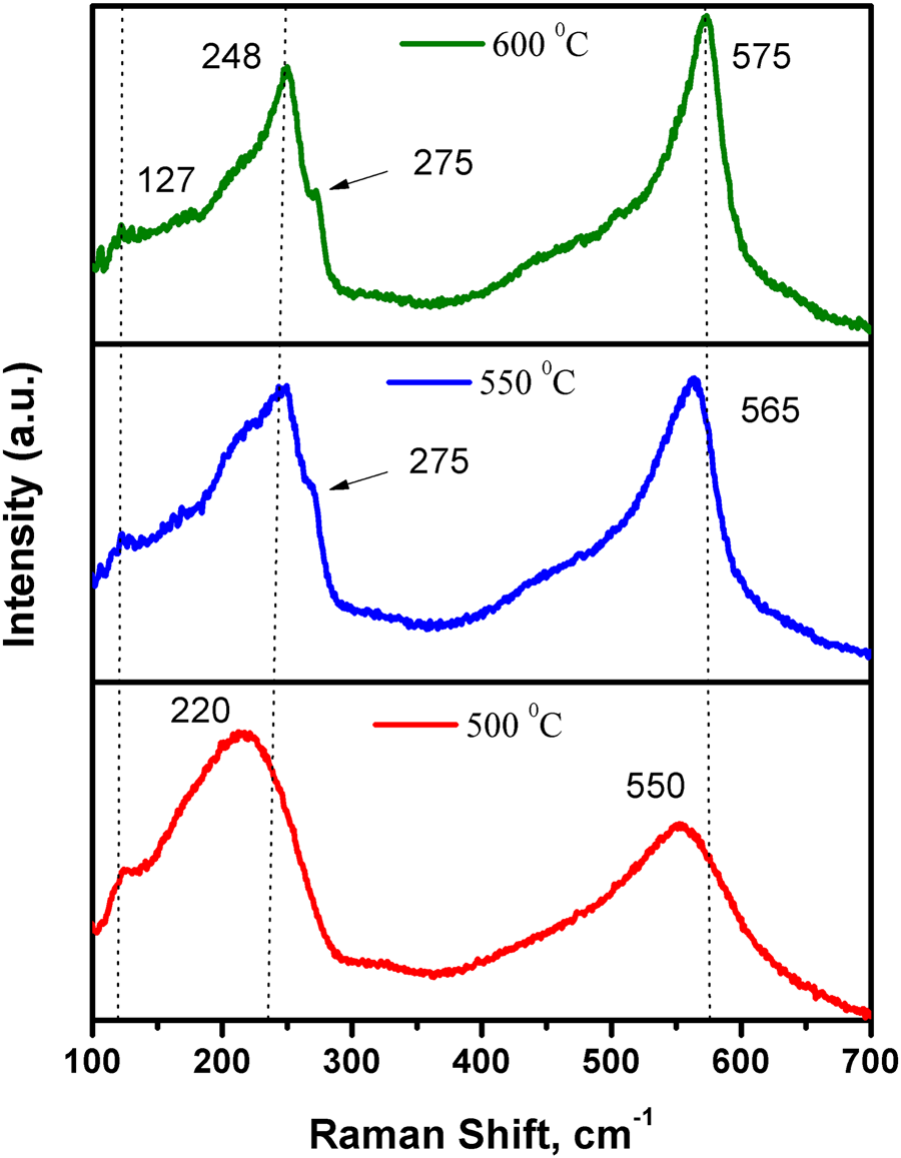
Raman spectra of the Zn(O,Se) films deposited in the substrate temperature region of 500–600 °C under nitrogen back-pressure.

RT Raman spectroscopy has also been used for further structural characterization of the deposited layers. Figure 2 displays the Raman spectra of Zn(O,Se) films deposited at 500–600 °C. All Zn(O,Se) layers deposited at 100–600 °C exhibited a weak band of Raman scattering located at 127 cm^−1^ which corresponds to Zn(O,Se) phase (see Supplementary Fig. S2 for the substrate temperature range 100–400 °C). In addition, Zn(O,Se) films, deposited in the temperature range from 100 to 500 °C, exhibited two strong broad bands located at 220 cm^−1^ and 550 cm^−1^, which correspond to the formation of Zn(O,Se) phase (amorphous in the range 100–400 °C). However, for Zn(O,Se) films deposited at 550 and 600 °C, an observable shift in both peaks position, to 248 cm^−1^ and (565/575 cm^−1^), can be seen, which originate from longitudinal optical (LO) phonon modes of ZnSe^17,38–41^ and longitudinal optical (LO) phonon E_1_ symmetry mode of ZnO^17,42–44^ respectively. These shifts in both Raman bands confirmed that ternary Zn(O,Se) polycrystalline phase formed at 500 °C, shifted towards formation of ZnSe(1-y)Oy/ZnO(1-z)Sez solid solutions, due to the decrease of O solubility in ZnSe and vice versa at 550/600 °C substrate temperature.

Besides those Raman bands, extra weak peaks started appearing, from 550 °C, located at 275 cm^−1^. This additional Raman scattering is attributed to the intrinsic host lattice defects, such as oxygen and selenium vacancies, or Zn interstitials, which become activated with high substrate temperatures 550 and 600 °C^45^.

**Figure 3:**
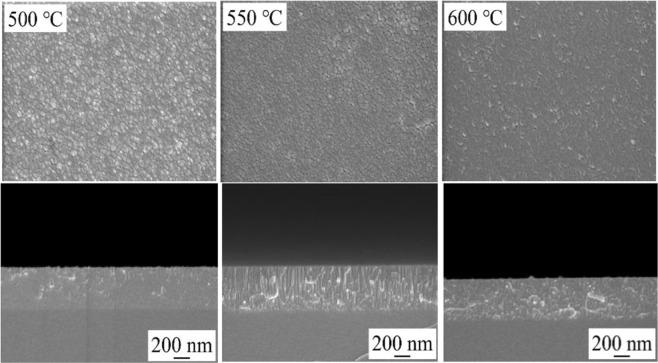
HR-SEM images and cross-sectional views of the Zn(O,Se) films deposited in the substrate temperature region of 500–600 °C under nitrogen back-pressure.

Figure 3 represents surface and cross-sectional SEM images of Zn(O,Se) films deposited at 500–600 °C. Homogenous, dense, and well-adherent to the glass substrate surface, the films have been grown over the entire temperature range. Moreover, with increasing the substrate temperature from RT to 500 °C the grain size of the films increased, with an average grain size between 15–86 nm, and from 550 °C and above, the grain size showed a decreasing tendency. Furthermore, as can be seen from the cross-sectional views in Fig. 3 and (Supplementary Fig. S3), the thickness of the deposited films decreased with increasing substrate temperature from 733 nm for the film deposited at RT to 454 nm for the film deposited at 500 °C, see Table 1 and (Supplementary Table S1). A similar trend has been observed in previous work of PLD Zn(O,Se) films deposited in high vacuum^16^, and was explained by the formation of a denser, compact and highly ordered polycrystalline structure, with the increase of substrate temperature.

Table 1 shows the atomic ratios of the elements in Zn(O,Se) films deposited at 500–600 °C (see Supplementary Table S1, for the temperature range RT – 400 °C). This shows stable ratios of the elemental contents in the whole substrate temperature range, accompanied with minor differences in the oxygen and selenium contents with changing the substrate temperature. When compared to PLD deposited Zn(O,Se) films in high vacuum^16^, the PLD deposited Zn(O,Se) films in nitrogen background pressure maintained stable elemental contents, particularly for Se content, in the whole range of the substrate temperature. This can be attributed to the positive effect of nitrogen background pressure which prevents evaporation of Se before the nucleation stage.

**Figure 4:**
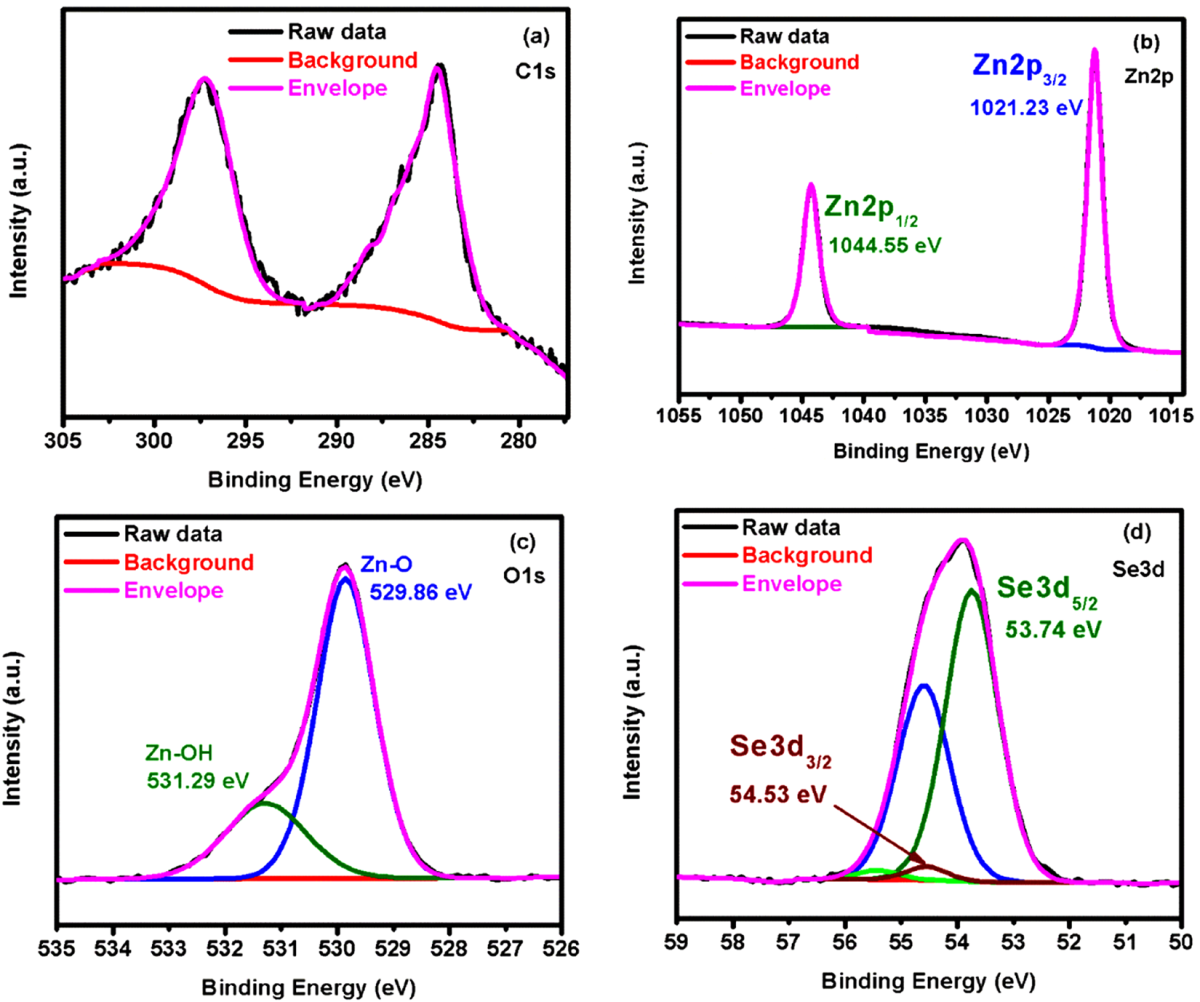
XPS spectrum of the Zn(O,Se) layers deposited at 500 °C under nitrogen back-pressure: (**a**) C 1s, (**b**) Zn 2p, (**c**) O 1s and (**d**) Se 3d.

High-resolution XPS was used to quantitatively analyse the surface chemical composition and to identify the possible phases of each species of the Zn(O,Se) layers deposited at 500–600 °C. XPS analysis has been conducted for all Zn(O,Se) samples 500–600 °C after surface cleaning with argon (Ar^+^) sputtering for 30s. Figure 4 shows the XPS spectra of C 1s, Zn 2p, O 1s, and Se 3d of Zn(O,Se) layers deposited at 500 °C. The XPS analysis revealed that the samples consisted of Zn, O, and Se, and no impurities were found. Specifically, Fig. 4a shows the binding energy for the adventitious C 1s peak at 284.6 eV which was considered for charge corrections, moreover, those two peaks appeared in C 1s spectrum positioned at 284.30 eV and 297.25 eV are attributed to Se Auger peaks. Figure 4b shows the Zn 2p XPS core-level spectrum of the sample deposited at 500 °C. Only one set of doublet was used to fit Zn 2p spectrum. None of fitting parameters, FWHM, area, nor doublet separation, were fixed. The spectra show features at 1021.23 eV and 1044.55 eV corresponding to Zn2p_3/2_ and Zn2p_1/2_, respectively. The spin-orbit splitting of 23.32 eV between the peaks is in a good agreement with the value for Zn^2+^ oxidation state^46–49^. For O1s two peaks of different forms of oxygen were fitted Fig. 4c. The first oxygen peak is positioned at the lower binding energy of 529.86 eV, and is assigned to O ions in the Zn–O bonding of the Zn(O,Se) layers^46–49^. The second peak located at 531.29 eV is related to OH group absorbed onto the surface of the Zn(O,Se) layers^47^. The Se 3d region shown in Fig. 4d displays two sets of doublets, first main peak centered at 53.74 eV binding energy and the other at 54.53 eV. Each doublet comprises two peaks due to the spin-orbit splitting of the Se 3d level, which are assigned to Se 3d_5/2_ and Se 3d_3/2_ of Zn-Se bonding^50^. The trend is similar for Zn(O,Se) layers deposited at 550 and 600 °C except minor shifts in binding energy, due to the formation of different solid solution of ZnSe(1-y)Oy and ZnO(1-z)Sez (see Supplementary Figs. S5 and S6). The elemental ratios of Zn(O,Se) layers were determined also on the basis of XPS surface analysis. Significant increase in Zn contents was observed with the increase in substrate temperature from 500 °C to 550/600 °C from 52 at% to 57 at%, while remarkable decrease was found for oxygen contents in similar temperature interval. Regardless, the selenium contents remain stable for the substrate temperature range 500–600 °C (see Supplementary Table S2). The modified Auger parameters (m-AP) α′ for Zn(O,Se) layers were calculated from the experimental binding energies of the Zn 2p_3/2_ and Se 3d_5/2_ photoelectron peaks and kinetic energies of Zn L_3_M_45_M_45_ and Se L_3_M_45_M_45_ Auger peak values^51^ of standard pure ZnO, ZnSe, SeO_2_ phases and Zn(O,Se) layers deposited at 500 °C are presented in Table 2. By considering Wanger plot^52,53^ and data presented in Table 2, we compared peak positions, kinetic energies and m-APs (α′) of Zn(O,Se) layers with standard values for pure ZnO, ZnSe and SeO_2_ phases^51–53^. Thus, the calculated three parameters of Zn(O,Se) layers are different from those standard values, which mean different compound has been formed, i.e. ternary Zn(O,Se) compound has been formed at 500 °C. In addition, core-level peak positions, Auger peak kinetic energies and m-APs (α′) of Zn(O,Se) layers deposited at 550/600 °C are different from that of Zn(O,Se) layers deposited at 500 °C (see Supplementary Tables S2), which mean formation of the solid solutions of ZnSe(1-y)Oy/ZnO(1-z)Sez in a good agreement with XRD and Raman results.

**Table 2:** Binding energy and modified Auger parameters (m-AP) for Zn(O,Se) layers deposited at 500 °C under nitrogen back-pressure.

Samples	Zn2p3/Se3d5	L_3_M_45_M_45_	m-AP	Refs.
	peak position	Kinetic energy	α′	
	eV	eV	eV	
ZnO	1022.10	987.70	2009.80	^50^
ZnO	1021.60	988.80	2010.40	^51^
ZnSe	1022.00	989.50	2011.50	^50^
Zn(O,Se), Zn2p	1021.23	989.30	2010.50	This work
SeO_2_	58.80	1301.60	1360.40	^50^
Zn(O,Se), Se3d	53.74	1306.58	1360.32	This work

Measurement error of XPS are (Binding energy ± 0.02 eV, for quantification ± 10%).

**Figure 5:**
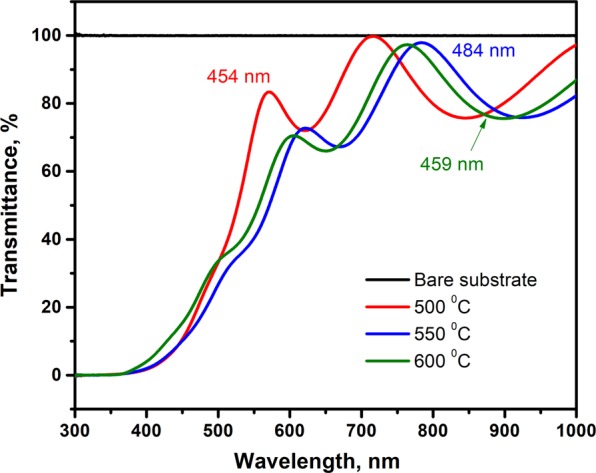
Transmittance spectra of bare substrate and Zn(O,Se) films deposited in the substrate temperature region of 500–600 °C under nitrogen back-pressure with their corresponding films thickness.

Figure 5 shows the optical transmittance spectra of Zn(O,Se) films deposited at 500–600 °C (see Supplementary Fig. S7 for the temperature range RT – 400 °C). All the produced films exhibited transparency of around 80% in the visible region of the spectra, except films produced at RT and 100 °C, which showed less than 80%. With increasing substrate temperature, the absorption edges shifted to a lower wavelength (blue shift). The optical band gaps of all Zn(O,Se) films were evaluated based on the Tauc relation (2)^7,54^ by plotting hv versus (αhv)^2^ and extrapolating the linear part of the plot to hv axis (x axis) (see Supplementary Fig. S8).

2 $$(\alpha hv)=A{(hv-{E}_{g})}^{n}$$

where α is absorption coefficient and estimated from α = 2.303 log (T/d), T is the transmittance, d is the film thickness, ${E}_{g}$ is the band gap, hv is the photon energy, eV is the unit for band gaps and exponent n depends on transition type (n = ½ for direct allowed).

The calculated band gaps of Zn(O,Se) films fluctuated with the substrate temperature from 2.76 eV for RT to 3.29 eV for 600 °C see Table 1 and (Supplementary Table S1 for Zn(O,Se) films deposited at RT – 400 °C). This fluctuation in the band gaps can be attributed to the variation in film thickness and shifting in the absorption edges with the substrate temperature. In addition, the films deposited at 550 and 600 °C showed two different band gaps ~2.98 and ~3.28 eV which is in excellent agreement with the XRD results which showed two diffraction peaks corresponding to a shift of Zn(O,Se) phase toward formation of ZnSe(1-y)Oy/ZnO(1-z)Sez solid solutions, as in XRD at 550/600 °C.

Table 3 shows the Hall effect measurements of Zn(O,Se) films deposited at 500–600 °C. All the films deposited below 500 °C showed very high dark resistivity, which was associated to their amorphous, semi-insulating, behaviour. The sign of the Hall coefficient has revealed that the electrons are the majority carriers in the PLD deposited Zn(O,Se) films, under nitrogen background pressure, which corresponds to the n-type conductivity of the films.

**Table 3:** The electrical parameters of the Zn(O,Se) films deposited in the temperature region of 500–600 °C under nitrogen back-pressure.

Ts, °C	Hall coefficient (cm^3^/C)^a^	Resistivity (Ω cm)^a^	Concentration (cm^−3^)^a^	Mobility (cm^2^/Vs)^a^
500	−2.4 × 10^7^	2.1 × 10^5^	3.0 × 10^11^	113.0
550	−6.0 × 10^6^	2.3 × 10^5^	1.0 × 10^12^	26.2
600	−2.0 × 10^2^	5.4 × 10^1^	3.4 × 10^16^	3.4

^a^Measurement errors of Hall effect measurement is ±5.0.

The films deposited at 500 °C were characterized as having high resistivity of 10^5^ Ω·cm, electron concentration of ~ 10^11^ cm^−3^ and a mobility of 113.0 cm^2^/V·s. By increasing the substrate temperature to 550 °C, the electron concentration increased by nearly one order of magnitude, from 10^11^ to 10^12^ cm^−3^, while the mobility was significantly reduced, up to 26.2 cm^2^/V·s. A further increase of the substrate temperature to 600 °C sharply increased the electron concentration by approximately four orders of magnitude (~10^16^ cm^−3^) and dramatically reduced the mobility to 3.4 cm^2^/V·s. The changes in the electrical properties of Zn(O,Se) films, grown under nitrogen background pressure, can be explained based on the same thermodynamic considerations from a previous study^16^. Therein, the Gibbs energies of the formation for ZnSe, ZnO and SeO_2_ in the temperature interval of 500–600 °C were analysed, as follows:

3 $$Z{n}_{(g)}+\frac{1}{2}{O}_{2(g)}\leftrightarrow Zn{O}_{(s)}(\Delta {G}^{{500}^{^\circ }C}=\mbox{--}74.76\,kcal/mole),$$

4 $$Z{n}_{(g)}+\frac{1}{2}S{e}_{2(g)}\leftrightarrow ZnS{e}_{(s)}(\Delta {G}^{{500}^{^\circ }C}=\mbox{--}51.33\,kcal/mole),$$

5 $$\frac{1}{2}S{e}_{2(g)}+{O}_{2(g)}\leftrightarrow Se{O}_{2(g)}\,(\Delta {G}^{{500}^{^\circ }C}=\mbox{--}30.97\,kcal/mole),$$

These thermodynamically favoured reactions indicate that the ZnO and ZnSe are the most stable phases in the Zn(O,Se) and together, with the formation of SeO_2_, generate conditions for increased partial pressure of oxygen and selenium above ZnO and ZnSe and hence the creation of a Zn-rich composition in the Zn(O,Se) vapour phase. This fact leads to an increase in the overall concentration of oxygen and selenium vacancies (n-type intrinsic defects), and consequently enhances the electron concentration in Zn(O,Se) films. As the electron concentration increased, with increasing substrate temperature, the Hall mobility decreased systematically (Table 3). This behaviour is explained by the scattering of carriers, through ionized oxygen/selenium vacancy defects with high concentrations, at elevated temperatures. The formation of Frenkel defects and the equilibrium constant in the Zn(O,Se) system are given below^16,55^.

6 $${\rm{Zn}}({{\rm{O}}}_{{\rm{x}}}{{\rm{Se}}}_{{\rm{y}}})\mathop{\to }\limits^{500-600^\circ {\rm{C}}}{[{{\rm{Zn}}}_{(1-{\rm{n}})}{{\rm{O}}}_{({\rm{x}}-2{\rm{n}})}{{\rm{Se}}}_{({\rm{y}}-{\rm{n}})}]}_{({\rm{s}})}\,+\,{{\rm{mSeO}}}_{2({\rm{g}})}\uparrow +\,{{\rm{nZn}}}_{({\rm{g}})}\,+\,(n-m){1/2}{{\rm{O}}}_{2({\rm{g}})}\,+\,(n-m){1/2}{{\rm{Se}}}_{2({\rm{g}})}$$

7 $$Zn{O}_{(s)}\leftrightarrow {[{{\rm{Zn}}}_{(1-{\rm{n}})}{{\rm{O}}}_{(1-{\rm{n}})}n{V}_{Zn}n{V}_{O}]}_{(s)}+{{\rm{nZn}}}_{({\rm{g}})}+{\rm{n}}\frac{1}{2}{{\rm{O}}}_{2({\rm{g}})}$$

8 $$ZnS{e}_{(s)}\leftrightarrow {[{{\rm{Zn}}}_{(1-{\rm{n}})}{{\rm{Se}}}_{(1-{\rm{n}})}n{V}_{Zn}n{V}_{Se}]}_{(s)}+{{\rm{nZn}}}_{({\rm{g}})}+{\rm{n}}\frac{1}{2}{{\rm{Se}}}_{2({\rm{g}})}$$

9 $${V}_{Zn}\leftrightarrow {V}_{Zn}^{-}+h\,\,\,\,({\rm{holes}}\,{\rm{donating}})$$

10 $${V}_{Se}\leftrightarrow {V}_{Se}^{+}+{e}^{-}\,\,\,\,({\rm{electrons}}\,{\rm{donating}})$$

11 $${V}_{O}\leftrightarrow {V}_{O}^{-}+{e}^{-}\,\,\,\,({\rm{electrons}}\,{\rm{donating}})$$

${K}_{(ZnO)}=[{V}_{Zn}^{-}]\cdot [{V}_{O}^{-}]$, where *K*_*(ZnO)*_ is the equilibrium constant for ZnO.

${K}_{(ZnSe)}=[{V}_{Zn}^{-}]\cdot [{V}_{Se}^{-}]$, where *K*_*(ZnSe)*_ is the equilibrium constant for ZnSe.

Thus, all these equations, and the aforementioned considerations, are valid for Zn(O,Se) films deposited under nitrogen atmosphere, and the background pressure only serves to inhibit the transport of the material, through the gas phase to the substrate, by reducing the mean free path and kinetic energy of the ablated particles. The latter phenomenon (i.e., reduced mean free path) has an impact of retaining the Se content in Zn(O,Se) system (EDX analysis showed constant Se concentration in the entire temperature interval).

Compared to previous work^16^, the electron concentration in the Zn(O,Se) films grown at 500–600 °C, in high vacuum, is significantly higher than the electron concentration in Zn(O,Se) films deposited at the same temperatures, but under nitrogen pressure, although for both growth conditions the trend is the same. Considering the relatively stable Se concentration (according to EDX) the reduced electron concentration can be explained as being due to slightly increased partial pressure of Se (in the form of SeO_2_) in the Zn(O,Se) system. According to Eq. (5) the higher the concentration of Se in the solid phase is, the higher the partial pressure of SeO_2_ above Zn(O,Se) and the lower the concentration of Se vacancies. The increased partial pressure of Se will also have an impact on ZnO (due to solubility of Se in ZnO) by reducing the concentration of oxygen vacancies. Under these conditions, the reduced concentrations of selenium and oxygen vacancies lowers the electron concentration in Zn(O,Se) grown under nitrogen atmosphere. The XRD results showed two diffraction peaks with an increase in the substrate temperature to 550 and 600 °C. This fact indicates, that at these temperatures, the solubility of oxygen in ZnSe, selenium in ZnO (i.e., ZnSe has low solubility in ZnO and vice versa) and solubility of SeO_2_ from the solid phase are significantly reduced and ZnSe and ZnO does not form a homogenous Zn(O,Se) solid solution. Thus, the changes in the electrical properties correlate well with XRD and SEM/EDX results.

In summary, homogenous, adherent and polycrystalline Zn(O,Se) layers have been deposited by PLD under 50 mTorr of nitrogen background pressure. All Zn(O,Se) layers deposited in the substrate temperature range RT – 400 °C were amorphous, and a conductive polycrystalline Zn(O,Se) phase was formed at 500 °C. Optical investigation confirmed that all films are practically transparent in the visible range of the spectrum with band gag value around 3 eV. It was found, that the nitrogen partial pressure had a dramatic influence on the defect structure of the deposited layers and maintained stable ratios of the elemental contents, over the whole substrate temperature range, in comparison with PLD of Zn(O,Se) films in high vacuum.

## Methods

### Deposition of Zn(O,Se) structure

Zn(O,Se) layers were deposited onto glass substrates by PLD under nitrogen background pressure in the temperature range from RT to 600 °C. Soda lime glass substrates, 3.6 × 3.6 cm in size, were prepared and ultrasonically cleaned for 15 min in 20% solution of Decon 90 and deionized water at 50 °C respectively. The substrates were dried under filtered air flow, then exposed to Nova Scan Digital UV-Ozone cleaning system for 15 min. (ZnO:ZnSe) of 25.4 mm dia. × 6 mm thick targets for PLD were prepared by hot-pressing of pre-mixed fine powders of ZnO (75 at %) and ZnSe (25 at %) (99.99% purity, Testbourne Ltd). The deposition was performed by Neocera Pioneer 120 PLD system equipped with 248 nm KrF excimer laser (Coherent Compex Pro 102 F). The target to substrate distance was 9 cm^16^. The background pressure in the vacuum chamber was 50 mTorr at a constant flow rate of nitrogen of 9 standard cubic centimeter (Sccm). For all substrate temperatures, similar deposition conditions were applied as follows: (40000 laser pulses, 200 mJ pulse energy, 10 Hz repetition rate), the laser beam was focused on ~5 mm^2^ spot on the surface of (ZnO: ZnSe) target. For comparison, pure phases of ZnO and ZnSe layers were also deposited by PLD at 500 °C. The substrate was rotated during the deposition process for uniform distribution of the ablated materials onto the substrate, and the target was rotating and rastering to avoid local heating of the target and uniform consumption of the materials.

### Characterization

The phase composition of the prepared Zn(O,Se) films was characterized by X-ray diffraction (XRD) and Raman spectroscopy. XRD analyses were conducted in the Bragg - Brentano (θ-2θ) geometry using a Rigaku Ultima IV diffractometer with Cu Kα radiation. Crystallite size was evaluated using the software PDXL Version 1.4.0.3 on the Rigaku system. Raman measurements were performed by a Horiba’s LabRam HR800 spectrometer, equipped with 532 nm green laser with a spot size of ~5 µm. Optical transmittance spectra were measured in the wavelength range of 300–1000 nm by means of Shimadzu UV-1800, UV-Vis spectrophotometer. Film thickness and morphology were characterized by means of high-resolution scanning electron microscope (HR-SEM) Zeiss Merlin equipped with In-Lens SE detector for topographic imaging. Elemental analysis was conducted using Energy Dispersive X-ray (EDX) system (Bruker EDX-XFlash6/30 detector)^7^. XPS measurements were performed with a Kratos Axis Ultra DLD XPS spectrometer with monochromatic Al K_α_ radiation (1486.6 eV). The pass energy for the high-resolution scan was kept at 20 eV. Scans were acquired with steps of 100 meV. A charge neutraliser system was switched on during acquisitions. The spectrometer was calibrated to give Au 4f_7/2_ metallic gold binding energy of 83.98 eV and the spectrometer dispersion is adjusted to give a binding energy of 932.68 eV for metallic Cu 2p_3/2_. The binding energy for the adventitious C 1s peak at 284.6 eV was considered for charge corrections. Mini Beam I Ar^+^ ion source was operated at 2 keV and 10 mA for 30 s to sputter clean the samples surface as they were exposed to the atmospheric conditions.

Hall measurements were performed by the Van Der Pauw method at room temperature using Ecopia HMS-3000 system. The gold spring probes were placed at the corners of the sample symmetrically. A constant current passed through the electrodes and an applied magnetic field was 0.55 T. Hall effect parameters, such as charge carrier’s mobility, concentration, Hall coefficient and resistivity were calculated.

## Supplementary information

Supplementary Information

## Data Availability

All data generated or analysed during this study are included in this published article (and its Supplementary Information files).
