# Supplementary material for: Pulsed laser deposition of Zn(O,Se) layers in nitrogen background Pressure
Source: Sci Rep. 2019 Nov 25;9:17443. doi: 10.1038/s41598-019-54008-1 (PMC6877616; doi:10.1038/s41598-019-54008-1)
Supplement: Supplementary file 1 — Supplementary Information [file 41598_2019_54008_MOESM1_ESM.pdf]

Pulsed laser deposition of Zn(O,Se) layers in nitrogen background pressure

Akram Abdalla[a,*], Sergei Bereznev[a], Nicolae Spalatu[a], Olga Volobujeva[a], Natalja Sleptsuk[b], and Mati Danilson[a]

[a] School of Engineering, Department of Materials and Environmental Technology, Tallinn University of Technology, Ehitajate tee 5, Tallinn 19086, Estonia.

[b] TJS Department of Electronics, Tallinn University of Technology, Ehitajate tee 5, Tallinn 19086, Estonia.

*corresponding author:

Akram Abdalla (email address: akrami.abdalla@gmail.com)

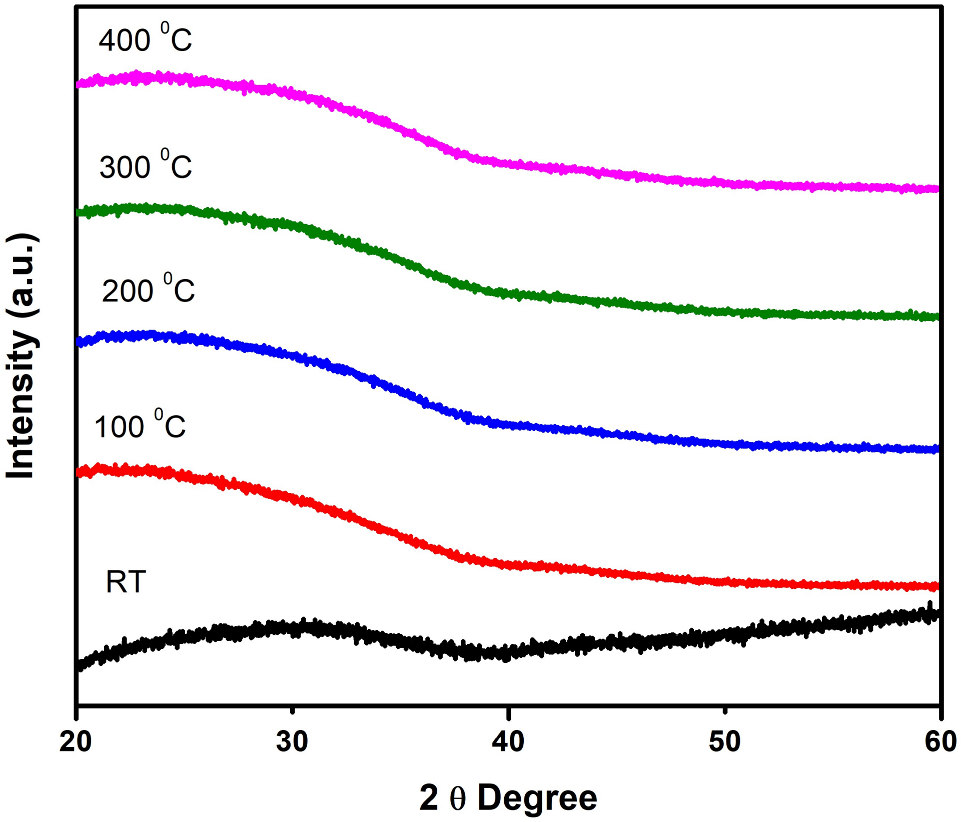

Figure S1: XRD patterns of the Zn(O,Se) films deposited in the substrate temperature region of RT – 400°C under nitrogen back-pressure.

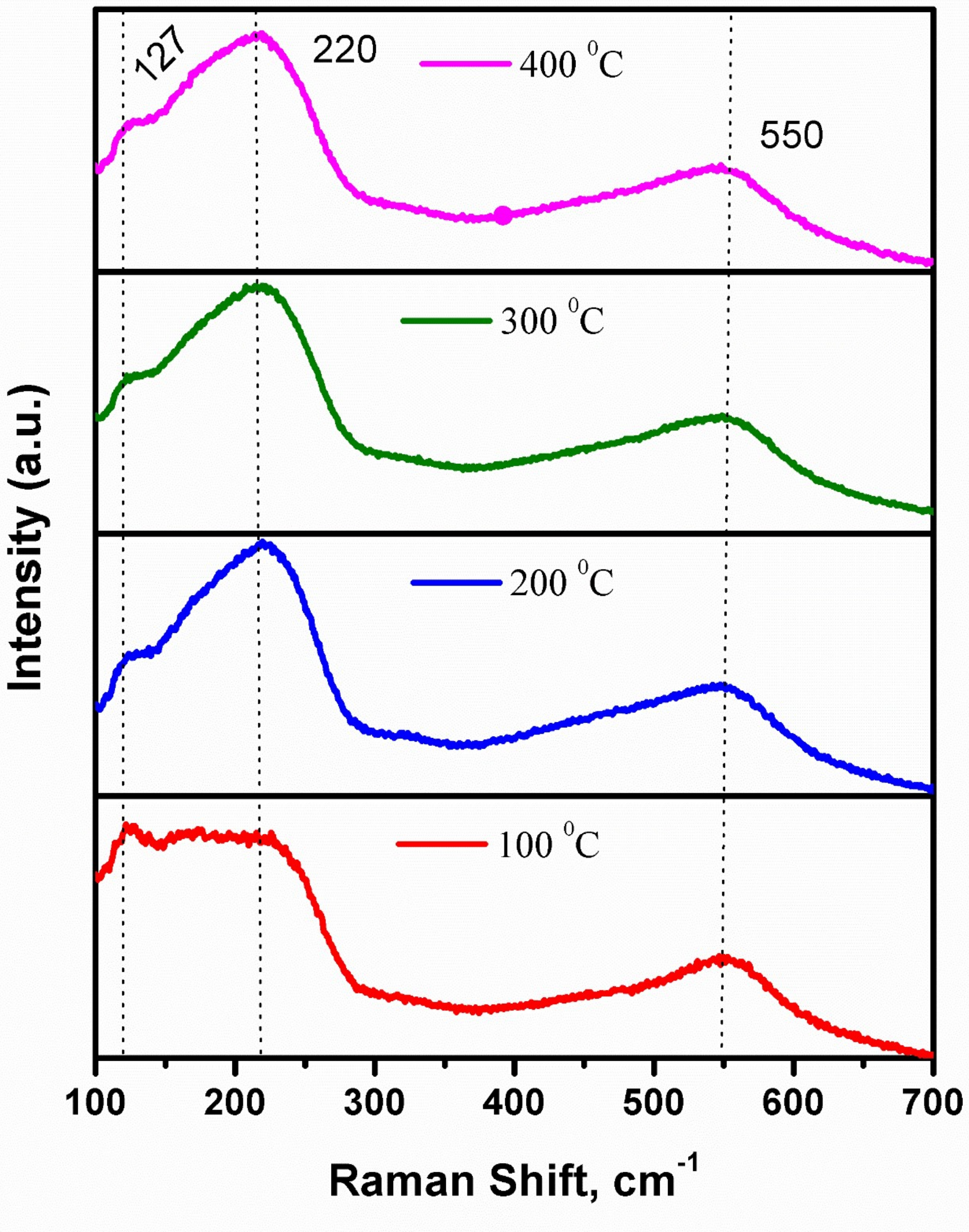

Figure S2: Raman spectra of the Zn(O,Se) films deposited in the substrate temperature region of 100 – 400°C under nitrogen back-pressure.

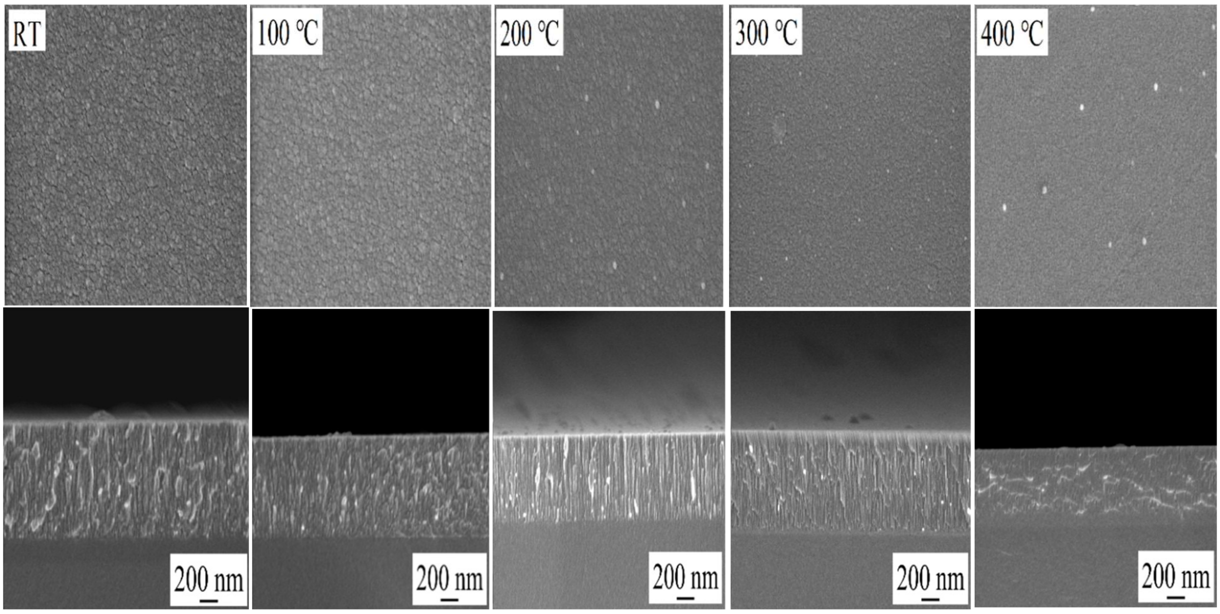

Figure S3: HR-SEM images and cross-sectional views of the Zn(O,Se) films deposited onto glass substrate at RT – 400°C under nitrogen back-pressure.

Table S1: The elemental contents, films thicknesses and band gaps values (E_g) of the Zn(O,Se) films deposited at RT – 400°C under nitrogen back-pressure.

Ts, (°C)	Elemental (at%)[a]			Thickness (nm)	Eg (eV)
	Zn	O	Se		
RT	50	39	11	733	2.76
100	50	38	12	639	2.98
200	50	36	14	554	3.00
300	50	36	14	647	2.80
400	50	36	14	512	2.83

[a] Measurement error of EDX is ± 0.5

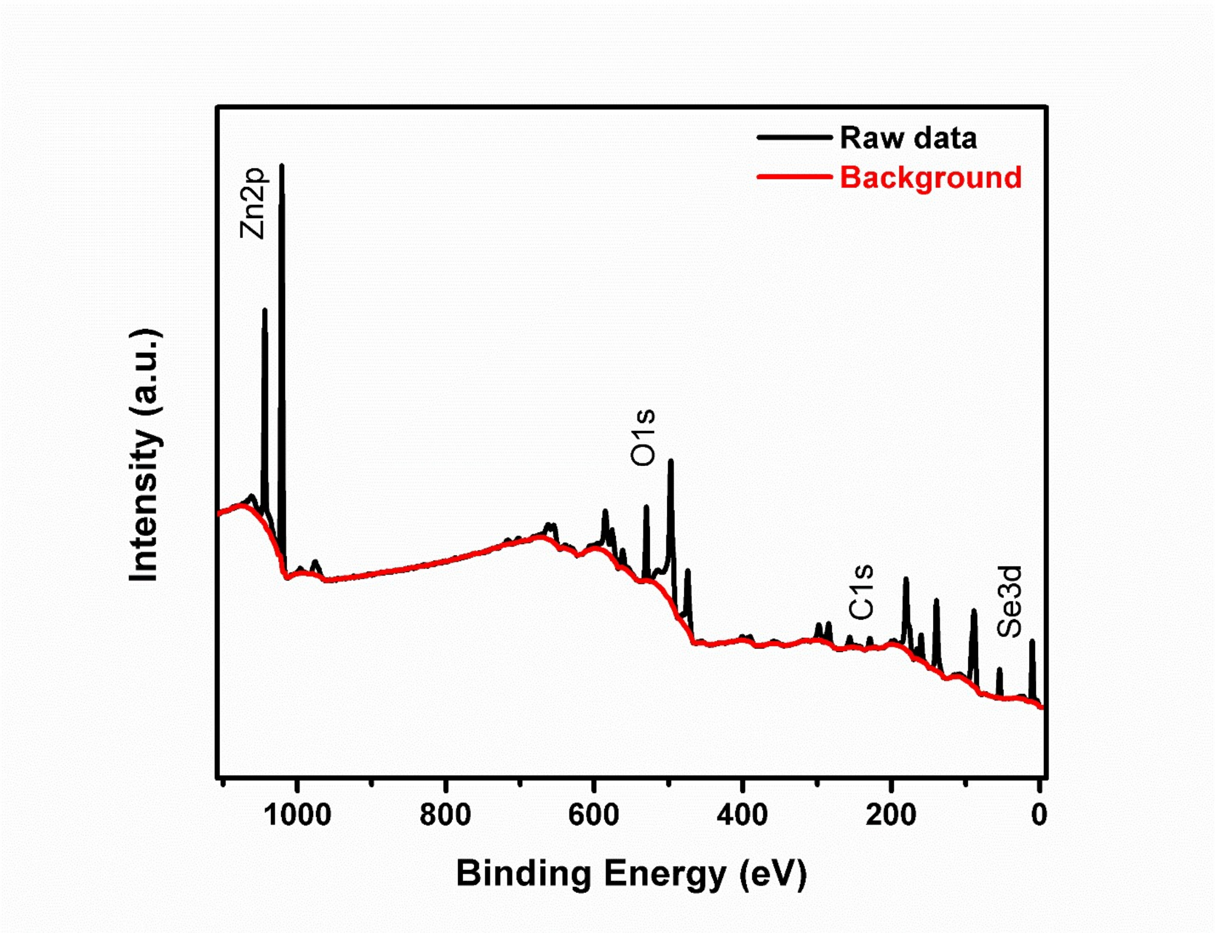

Figure S4: XPS wide survey spectra of the Zn(O,Se) layers deposited at 500°C under nitrogen back-pressure including all C 1s, Zn 2p, O 1s and Se 3d phases.

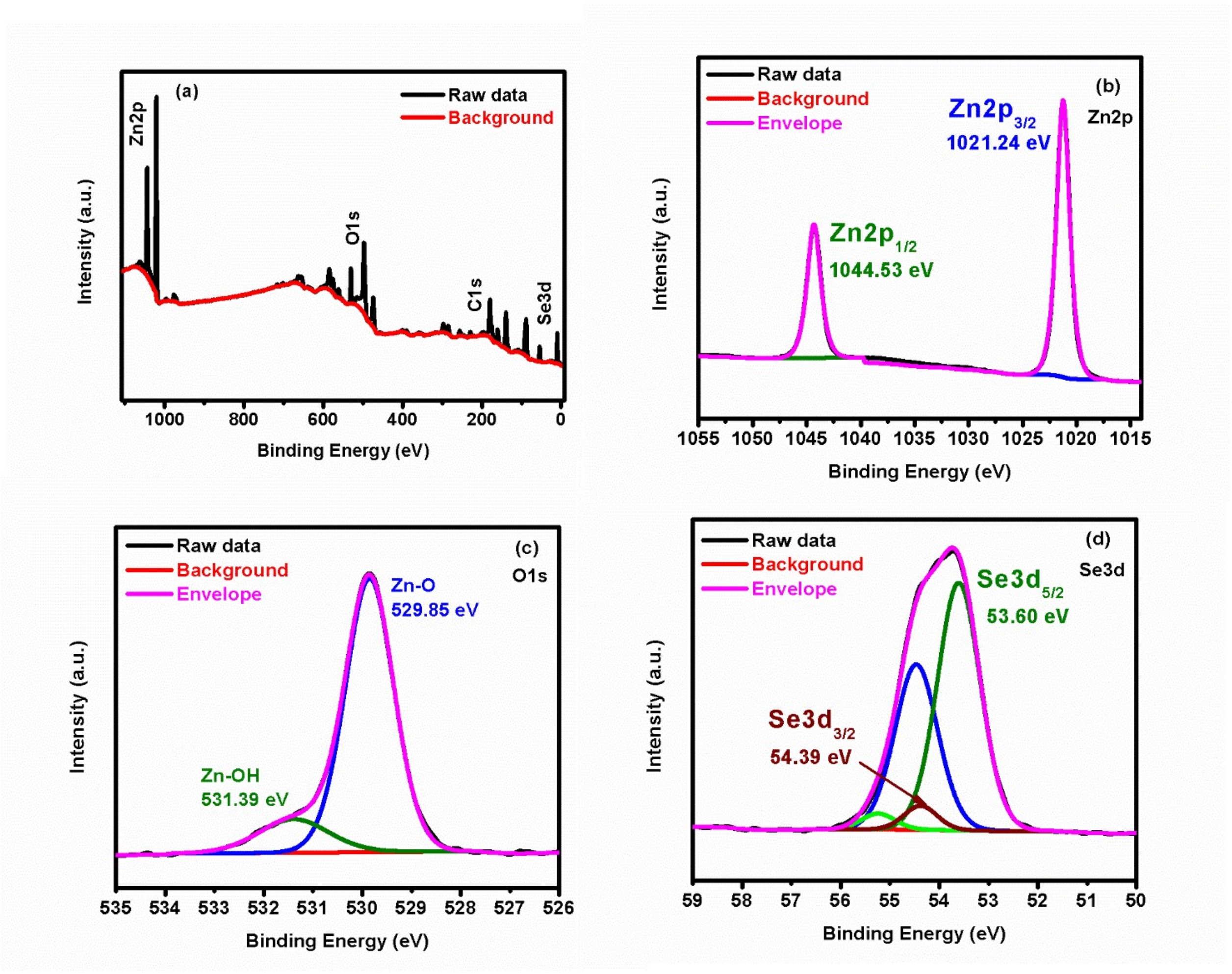

Figure S5: XPS spectrum of the Zn(O,Se) layers deposited at 550°C C under nitrogen back-pressure: (a) wide survey spectrum, (b) Zn 2p, (c) O 1s and (d) Se 3d.

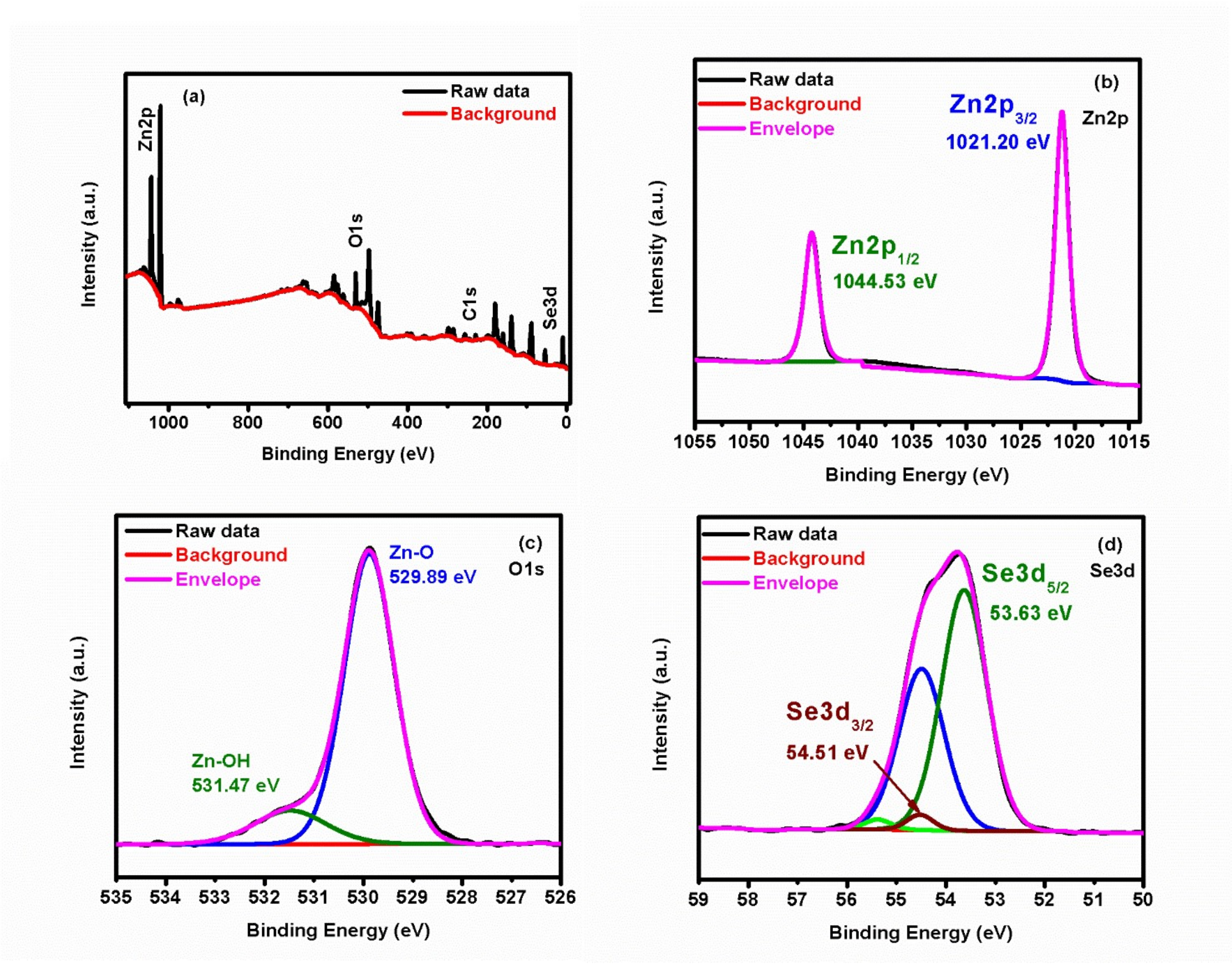

Figure S6: XPS spectrum of the Zn(O,Se) layers deposited at 600°C C under nitrogen back-pressure: (a) wide survey spectrum, (b) Zn 2p, (c) O 1s and (d) Se 3d.

Table S2: The modified Auger parameters and elemental contents (XPS based) of the Zn(O,Se) films deposited at 500 – 600°C under nitrogen back-pressure.

Ts	positions (eV)	positions (eV)	$L_3M_{45}M_{45}$ K.E (eV)	$L_3M_{45}M_{45}$ K.E (eV)	m-AP α′ (eV)	m-AP α′ (eV)	Ratios at%		
°C	Zn 2p	Se 3d	Zn 2p	Se 3d	Zn 2p	Se 3d	Zn	O	Se
500	1021.23	53.74	989.27	1306.58	2010.50	1360.32	52	37	11
550	1021.24	53.60	989.38	1306.78	2010.63	1360.38	57	32	11
600	1021.20	53.63	989.41	1306.86	2010.61	1360.50	57	32	11

Measurement error of XPS are (Binding energy ± 0.02 eV, for quantification ± 10%)

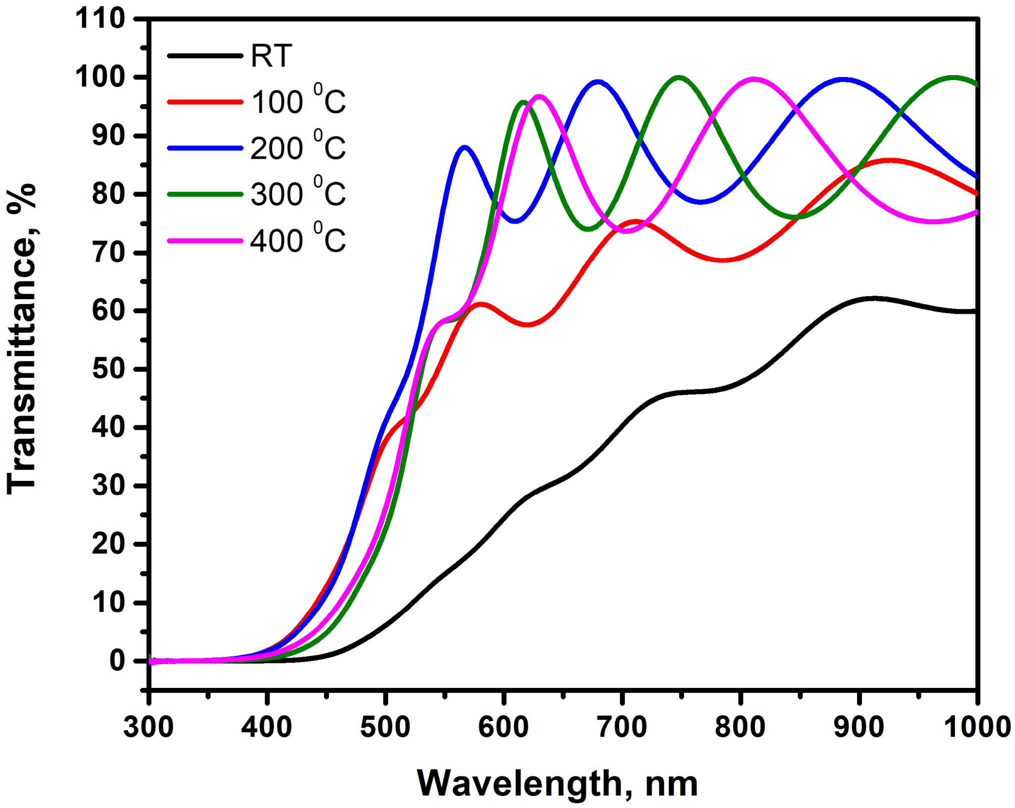

Figure S7: Transmittance spectra of Zn(O,Se) films deposited in the substrate temperature region of RT – 400°C under nitrogen back-pressure.

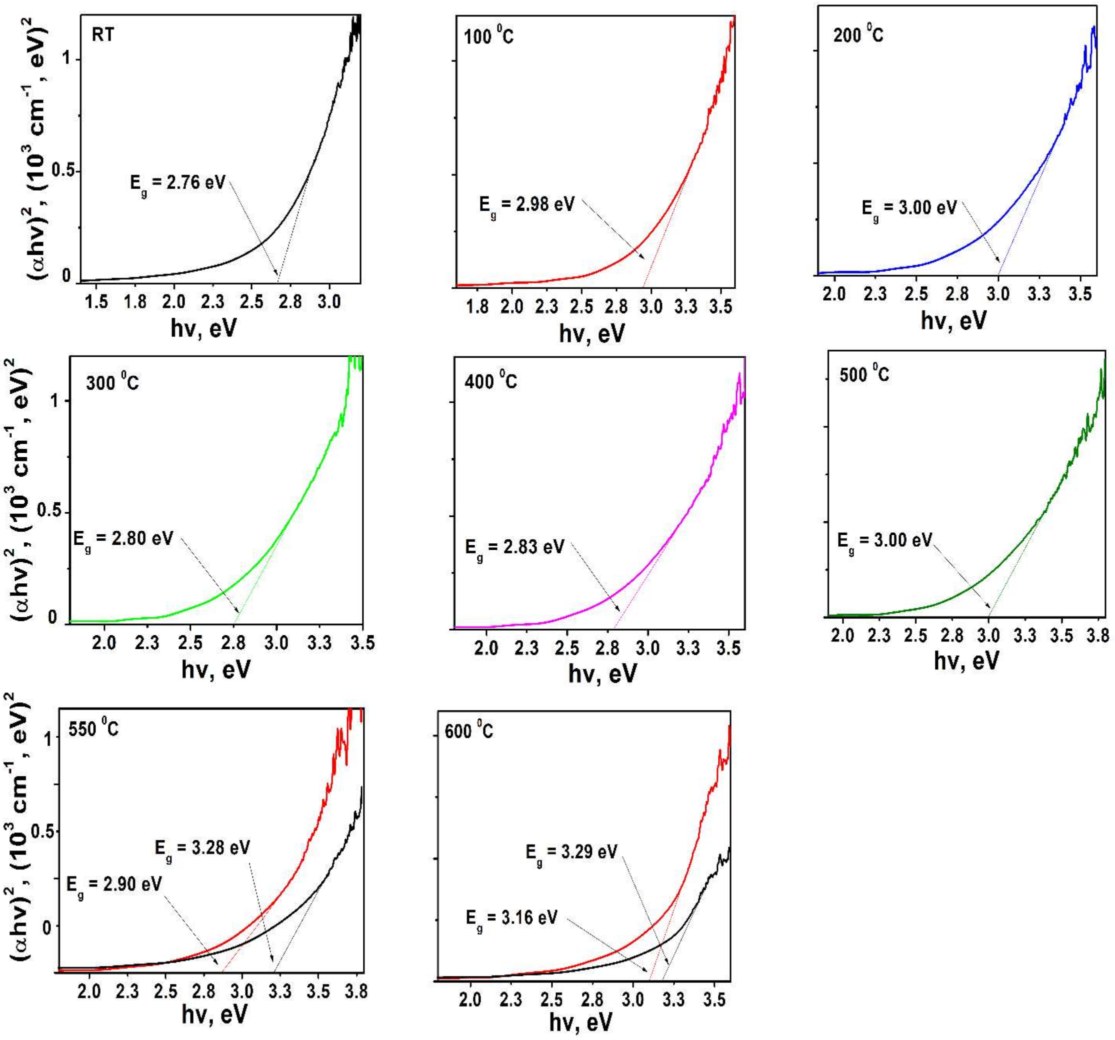

Figure S8:Tauc plots of Zn(O,Se) films deposited in the substrate temperature region of RT – 600℃ under nitrogen back-pressure.
